# Comparative analysis of *MTP* -493G/T and *ABCG2* 34G/A polymorphisms and theirs expression in HIV-associated lipodystrophy patients

**DOI:** 10.3389/fcvm.2023.1177054

**Published:** 2023-05-30

**Authors:** HariOm Singh, Chandrashekhar Jori, Shyamveer, Supriya D. Mahajan, Ravikumar Aalinkeel, Kathiravan Kaliyappan, Stanley A. Schwartz, Meenakshi Bhattacharya, Ruhi Shaikh, Madhukar Salve, Jyoti Deshmukh, Nemat Ali, Mohammad Khalid Parvez

**Affiliations:** ^1^ Department of Molecular Biology, National AIDS Research Institute, Pune, India; ^2^ Department of Medicine, Jacobs School of Medicine & Biomedical Sciences, University at Buffalo’s Clinical Translational Research Center, Buffalo, NY, United States; ^3^ Department of Medicine, Government Medical College & Hospital, Aurangabad, India; ^4^ ART Plus Centre, OPD-136, Government Medical College & Hospital, Aurangabad, India; ^5^ Department of Pharmacology and Toxicology, College of Pharmacy, King Saud University, Riyadh, Saudi Arabia; ^6^ Department of Pharmacognosy, College of Pharmacy, King Saud University, Riyadh, Saudi Arabia

**Keywords:** genetic predisposition, *MTP*, *ABCG2*, HIVLD, association studies

## Abstract

HIV-associated lipodystrophy (HIVLD) is a metabolic condition with an irregularity in the production of lipoprotein particles, and its occurrence varies among HIV-infected patients. *MTP* and *ABCG2* genes have a role in the transport of lipoproteins. The polymorphisms of *MTP* -493G/T and *ABCG2* 34G/A affect its expression and influence the secretion and transportation of lipoproteins. Hence, we investigated the *MTP -*493G/T and *ABCG2* 34G/A polymorphisms in 187 HIV-infected patients (64 with HIVLD and 123 without HIVLD) along with 139 healthy controls using polymerase chain reaction (PCR)-restriction fragment length polymorphism and expression analysis using real-time PCR. *ABCG2* 34A allele showed an insignificantly reduced risk of LDHIV severity [*P* = 0.07, odds ratio (OR) = 0.55]. *MTP* -493T allele exhibited a non-significantly reduced risk for the development of dyslipidemia (*P* = 0.08, OR = 0.71). In patients with HIVLD*, the ABCG2* 34GA genotype was linked with impaired low-density lipoprotein levels and showed a reduced risk for LDHIV severity (*P* = 0.04, OR = 0.17). In patients without HIVLD, *the ABCG2* 34GA genotype was associated with impaired triglyceride levels with marginal significance and showed an increased risk for the development of dyslipidemia (*P* = 0.07, OR = 2.76). The expression level of *MTP* gene was 1.22-fold decreased in patients without HIVLD compared with that in patients with HIVLD. *ABCG2* gene was upregulated 2.16-fold in patients with HIVLD than in patients without HIVLD. In conclusion, *MTP* -493C/T polymorphism influences the expression level of *MTP* in patients without HIVLD. Individuals without HIVLD having *ABCG2* 34GA genotype with impaired triglyceride levels may facilitate dyslipidemia risk.

## Highlights

1. *MTP* -493C/T polymorphisms influence the expression level.
2. Individuals who have impaired triglyceride level with *ABCG2* 34GA genotype were likely to be associated with the risk of dyslipidemia.
3. Individuals who have impaired LDL, HDL, triglyceride, and cholesterol levels with *MTP* -493GT and *MTP* -493TT and *ABCG2* 234AA genotypes showed a trend of the risk for severity of LDHIV and dyslipidemia, respectively.

## Introduction

Though highly active antiretroviral therapy (*HAART*) has prominently decreased the rate of mortality in people living with HIV (PLWH), lipodystrophy in HIV-infected patients (LDHIV) and its metabolic complications remain a significant concern. HIV-associated lipodystrophy (HIVLD) usually develops into lipohypertrophy, lipoatrophy, or a combination of both in PLWH undergoing antiretroviral therapy (ART) and is associated with a higher risk for cardiovascular disease (CVD) (1). Dyslipidemia is a prominent metabolic condition that can occur due to irregularities in the production, processing, and degradation of lipoprotein particles. Literature reported that the occurrence of lipoatrophy varies from 13.3% to 52.9% (2–5). In India, the prevalence of lipodystrophy (LD) varies from 22% to 60.7% (6, 7). The effects of HIV and antiretroviral therapies on fat differ between individuals. However, studies reported that HIV-infected individuals who were not treated with ART had changes in their body fat composition (8–10). Variations in the effect of ART drugs among people have been probably linked to the genetic profiles of patients (11). Host genetic factors also play a role in producing these syndromes (12).

Microsomal triglyceride transfer protein (MTP) plays a role in the transportation of lipid molecules, primarily present in hepatocytes and enterocytes (13). It is necessary for the synthesis and secretion of ApoB-containing lipoproteins from the hepatocytes and enterocytes. The secretory pattern of ApoB-containing lipoproteins most probably affects the plasma low-density lipoprotein (LDL) and very-low-density lipoprotein (VLDL) levels (14). MTP plays an important role in transferring cholesterol esters (15) and triglycerides (TG) from the endoplasmic reticulum membranes to nascent ApoB lipoproteins, producing VLDL and removing lipids from the hepatocyte (16). *The MTP* gene is situated on chromosome 4q23. Genetic variations in the *MTP* gene affect the *MTP* expression and influence the secretion of lipoproteins in ER (17). Numerous single nucleotide polymorphisms (SNPs) in the *MTP* gene are known, and the promoter region -493G/T (rs1800591) polymorphism is one of the most commonly examined (18, 19). The G allele of *MTP* -493G/T polymorphism leads to decreased *MTP* transcription activity than the T allele, decreased hepatocyte triglyceride export, and increased intracellular TG accumulation (14, 20). Less manganese-superoxide dismutase (*MnSOD*) is transported to the mitochondria when the -493T allele is present in the mitochondrial targeting sequence (21). The MTP -493G/T polymorphism was linked to altered MTP gene expression and led to abnormal changes in export of lipids, causing deregulated hepatic lipid metabolism (22, 23). Non-alcoholic fatty liver disease risk was increased by the MTP -493G/T polymorphism (23).

ATP-binding cassette transporter G2 (*ABCG2*) protein belongs to the family of ABC transporters which is also called as breast cancer resistance protein (BCRP). It is involved in the transport of bile, lipoprotein, drug substrates, and other peptides (24). *ABCG2* is widely expressed in healthy cells and organs, including the liver, kidney, and testes, as well as in the gastrointestinal tract, hematopoietic stem cells, and capillary endothelial cells. *ABCG2* has a protective role in preventing the buildup of toxic xenobiotics in cells and organs (25). The overexpression of *ABCG2* leads to an inter-individual differences in the bioavailability of drugs (26).

The *ABCG2* gene is situated on chromosome 4q22; consisting of 16 exons encoding 655 amino acid residues which form a 72-kD membrane protein (27, 28). The *ABCG2* gene is highly polymorphic. Numerous allelic variants in *the ABCG2* gene are known; some of them may have an impact on the protein's function and/or expression which may also alter the transporter activity (29, 30). There are two significant functional variations among these *ABCG2* 34G/A (rs2231137, V12M), resulting in the substitution of Val12Met and *ABCG2* 421C/A (rs2231142, Q141K), causing a Glu141Lys substitution which were described and demonstrated to be associated with the negative effects of multiple drugs transported via *ABCG2* (31, 32). These polymorphisms are linked to lower *ABCG2* expression and protein production, which consequently results in decreased transporter activity (30, 32–34).

Until now, the report has not been published on the association of *MTP* -493G/T and *ABCG2* 34G/A polymorphisms with HIVLD worldwide. Hence, we evaluated the association of *MTP* -493G/T and *ABCG2* 34G/A polymorphisms with LDHIV along with its occurrence in patients without HIVLD and in healthy individuals.

## Materials and methods

### Subjects

This was a cross-sectional study. A total of 187 participants (HIV-infected individuals) were recruited from the OPD of Medicine Department, ART Plus Centre, Government Medical College (GMC) & Hospital, Aurangabad, Maharashtra, between 1 April 2021, and 1 May 2022. Out of 187 patients, a total 64 had HIVLD and show clinical evidence of lipodystrophy/lipoatrophy/dyslipidemia enrolled as *cases*. Clinical examinations of lipoatrophy/lipohypertrophy were done by experienced doctors (i.e., physicians) at the ART Plus Centre, GMC & Hospital, during routine clinic visits. The following requirements were taken care of while recruiting subjects for case studies: (a) HIV-1-infected patients who have been on ART for a prolonged period, for a minimum of 1 year; (b) HIV-1/AIDS patients suffering from metabolic disorder dyslipidemia, as determined by a study of lipid profile subsequent to a 14-h fast, and who had (i) triglyceride levels greater than 300 mg/dl, (ii) high-density lipoprotein (HDL) levels decreased below 35 mg/dl, (iii) LDL levels greater than 120 mg/dl, and (iv) cholesterol levels greater than 240 mg; and (c) HIV/AIDS patients with clinical signs of lipodystrophy and lipoatrophy. The following exclusion criteria were taken care of while recruiting subjects for case studies: (i) patients with HIV-1 infection who also have co-existing conditions such as tuberculosis (TB), diabetes mellitus, hepatitis B or C, autoimmune diseases, cancers, or cardiovascular disease history and (ii) patients with HIV-1 infection who regularly consume alcohol or recreational drug.

Out of 187 patients, a total of 123 were without HIVLD and enrolled as controls. The criteria for including participants in this group are as follows: (1) HIV-1-infected patients who were not related to dyslipidemia, lipoatrophy, or lipodystrophy syndrome, (2) gender and age were compatible, and (3) HIV-1-infected patients being treated with ART for at least 1 year. The exclusion criteria were the following: (1) patients with lipoatrophy or dyslipidemia; (2) HIV-1-infected patients who also have other illnesses such as cancer, diabetes mellitus, hepatitis B or C, TB, or any autoimmune disease; (3) patients who regularly consume alcohol; and (4) patients who indulged in recreational drug use.

The patients’ CD4 counts, blood glucose levels, and lipid profiles were measured routinely during clinic visits. A thorough clinical history after infection and before ART treatment, information related to current ART regimen given for treatment, and data on its duration were collected from each patient recruited for the study. A total of 139 individuals (those from the same family were excluded), free from HIV, hepatitis B and C and tuberculosis, age matched, and serum negative from HIV-ELISA test, were recruited as healthy controls. The institutional ethics committees (NARI) had approved the study, and a written informed consent from each participant was recorded.

Lipoatrophy was categorized as subcutaneous fat loss in one or more of the following regions: face (gaunt face and sunken eyes), buttocks, and limbs (skinny with obvious veins, muscle, or bones), whereas lipohypertrophy was characterized as vascular fat gain in at least one of the following areas: the trunk (wider waist circumference), neck or back base (buffalo hump), and the breasts. Dyslipidemia was diagnosed through the analysis of lipid profile, which includes triglycerides, LDL, cholesterol, and HDL-C.

### DNA extraction

Blood sample (2 ml) was withdrawn from recruited patients and kept at −8°C. Genomic DNA was extracted according to the given kit instructions from the pellets of peripheral blood samples using the QIAamp DNA Blood Mini Kit.

### Genotyping

Polymerase chain reaction (PCR) and restriction fragment length polymorphism (PCR-RFLP) technique was utilized to genotype *MTP* -493G/T and *ABCG2* 34G/A polymorphisms in recruited subjects for each group. The *MTP* -493G/T and *ABCG2* 34G/A polymorphism primers were used as specified for particular gene amplification (14, 35). For 25μl PCR reaction, 10pmol FP and RP (primers), 10 mM dNTPs mix, 1 unit Taq DNA polymerase (Bangalore Genei, India), 100 mM Tris-HCl, and 1.5 mM MgCl2 containing PCR reaction buffer, 100–150 ng genomic DNA was taken as the standard for amplification of genes. The following PCR conditions were used for *MTP* -493G/T: initial denaturation temperature at 95°C for 5 min, 30 cycles of denaturation were at 95°C for 30 s, annealing temperature at 59°C for 30 s, extension at 72°C for 45 s, and a final extension at 72°C for 7 min. For *ABCG2* 34G/A, the following PCR conditions were used: initial denaturation temperature at 94°C for 5 min, 35 cycles of denaturation at 94°C for 45 s, annealing temperature at 60°C for 45 s, extension at 72°C for 1 min, and a final extension at 72°C for 7 min. The restriction enzymes *HphI* and *BseMI* (MBI Fermentas Inc., Glen Burnie, MD, United States) were used to digest the amplified products of *MTP* and *ABCG2*, respectively. Genotyping after restriction digestion of *MTP* and *ABCG2* was performed on 15% of polyacrylamide gel using molecular weight markers and visualized after staining with ethidium bromide (EtBr). On the basis of sequence and location of SNP, genotypes of *MTP* -493G/T and *ABCG2* 34G/A were assigned as follows: for *MTP*: 109 bp for TT genotype; 109 bp, 89 bp, and 20 bp for GT genotype; and 109 bp and 89 bp for GG genotype; and for *ABCG2*: 291 bp for GG genotype, 291 bp, 261 bp, and 30 bp for GA genotype; and 261 bp and 30 bp for AA genotype. Veriti 96-well Thermal Cycler (Applied Biosystems, United States) was utilized to perform all the reaction. In a 2% agarose gel, PCR products were run with molecular weight markers and visualized the band with EtBr staining. Twenty percent of the samples were re-genotyped by other laboratory personnel to avoid differences in genotyping, and 10% of the samples underwent sequencing in order to prevent genotyping error.

### qPCR analysis

For qPCR analysis, the oligonucleotide primers flanking a region of interest and DNA polymerase enzymes were used to amplify the sequence (Table 9 showed the sequence of the primer in the Supplementary material). The selective amplification and quantitative detection of *MTP* and *ABCG2* genes were performed using a qPCR machine. The qPCR machine was connected to a computer that maintains a record of fluorimeter output and uses software to analyze the experiment's results employing user-defined data, such as control wells and standards. The qPCR assay was carried out in 96-well plates by using 7,500 fast real-time PCR equipment (Applied Biosystems, CA, United States). The qPCR reaction was conducted in a total of 24 μl reaction volume with 10 μl DNA (10–100 ng of genomic DNA) and 12 μl of master mix cyber green, with each primer containing 2 μl.

### Data analysis

The mean ± standard deviation (SD) values were used to represent the age variable. Chi-square goodness-of-fit test was used to see if any healthy control subjects deviate from the Hardy–Weinberg equilibrium. To assess the genotype distribution in patients with and without HIVLD, as well as in patients without HIVLD compared to the healthy controls, *χ*^2^ statistics (Fisher's exact test for a cell size of <5) was utilized. Unconditional binary logistic regression was used to compute odds ratios (ORs) and 95% confidence interval (CI). The statistical analysis was conducted by using SPSS software version 17.0 (SPSS Inc., Released 2008; SPSS Statistics for Windows, Version 17.0, SPSS Inc., Chicago, IL, United States), and tests of statistical significance were two-sided and considered significant when a *P*-value was 0.05 (SPSS Inc., 2008). The qPCR data were analyzed on GraphPad prism directly using quantification Ct values, and the bar diagram was made in an Excel sheet.

## Results

The mean ages of the patients with HIVLD, the patients without HIVLD, and the healthy controls (years ± SD) were 38.37 ± 6.34 years, 37.87 ± 6.35 years, and 32.35 ± 7.45 years, respectively. Characteristics of the recruited patients with and without HIVLD along with the healthy controls are presented in Table 1.

**Table 1:** Characteristics of patients with HIVLD, patients without HIVLD, and healthy controls.

Subjects	Patients with HIVLD	Patients without HIVLD	Healthy controls
Total number	64	123	139
Mean age and standard deviation (years ± SD)	38.37 ± 6.34	37.87 ± 6.35	32.35 ± 7.45
Females	32 (50.79%)	64 (50.03%)	76 (45.3%)
Males	31 (49.20%)	59 (47.96%)	63 (54.7%)
Ethnicity	Western India	Western India	Western India
Cholesterol status (mg/dl))			
Normal (<200)	48 (75.0%)	77 (62.6%)	
Borderline high (200–239)	9 (14.1%)	27 (22.0%)	
High (>240)	7 (10.9%)	19 (15.4%)	
Triglyceride status (mg/dl)			
Normal (<150)	34 (53.1%)	75 (61.0%)	
Borderline high (150–199)	14 (21.9%)	26 (21.1%)	
High (240–499)	16 (25.0%)	22 (17.9%)	
LDL status (mg/dl)			
Normal (100–129)	43 (67.2%)	85 (69.1%)	
Borderline high (130–159)	11 (17.2%)	22 (17.9%)	
High (160–189)	10 (15.6%)	16 (13.0%)	
HDL status (mg/dl)			
Normal (>60)	5 (7.8%)	20 (16.3%)	
Borderline low (41–60)	41 (64.1%)	67 (54.5%)	
Very low (<40)	18 (28.1%)	36 (29.3%)	
Fasting glucose status (mg/dl)			
Normal (<100)	57 (89.1%)	99 (80.5%)	
Borderline/prediabetic high (100–125)	5 (7.8%)	13 (10.6%)	
High/diabetic (>126)	2 (3.1%)	11 (10.6%)	

HIVLD, HIV-associated lipodystrophy.

### *MTP* -493G/T and *ABCG2* 34G/A polymorphisms and HIVLD

The genotype and allele frequencies of *MTP* -493G/T and *ABCG2* 34G**/**A polymorphisms in patients with HIVLD, patients without HIVLD, and healthy controls are shown in Table 2. *MTP -*493GG, *MTP* -493GT, and *MTP* -493TT and *ABCG2* 34GG, *ABCG2* 34GA, and *ABCG2* 34AA genotypes were distributed almost similarly between patients with and without HIVLD and patients with HIVLD and healthy controls (43.8% vs. 51.2%, 40.6% vs. 36.6%, 15.6% vs. 12.2%, 78.1% vs. 66.7%, 20.3% vs. 27.6%, and 1.6% vs. 5.7% and 43.8% vs. 43.9%, 40.6% vs. 36.0%, 15.6% vs. 20.1%, 78.1% vs. 65.5%, 65.5% vs. 31.7%, 1.6% vs. 2.9%). *ABCG2* 34A allele was dispersed dissimilarly between patients with and without HIVLD and showed a reduced risk for severity of LDHIV with borderline significance **(**11.71% vs. 19.51%; *P* = 0.07, OR = 0.55, 95% CI 0.28–1.06).

**Table 2:** Frequency distribution of *MTP -*493G/T and *ABCG2* 34G/A polymorphisms in patients with HIVLD, patients without HIVLD, and healthy controls.

Genotypes *MTP -*493G/T	Patients with HIVLD, *N* = 64 (%)	Patients without HIVLD, *N* = 123 (%)	*P*-value	OR (95% CI)
GG	28 (43.8%)	63 (51.2%)	1	Reference
GT	26 (40.6%)	45 (36.6%)	0.32	1.64 (0.61–4.45)
TT	10 (15.6%)	15 (12.2%)	0.60	1.30 (0.47–3.61)
Alleles *MTP -*493G/T	Patients with HIVLD, *N* = 128 (%)	Patients without HIVLD, *N* = 246 (%)	*P*-value	OR (95% CI)
G	82 (63%)	171 (61%)	1	Reference
T	46 (37%)	75 (39%)	0.34	1.28 (0.79–2.06)
Genotypes *ABCG2* 34G/A	Patients with HIVLD, *N* = 64 (%)	Patients without HIVLD, *N* = 123 (%)	*P*-value	OR (95% CI)
GG	50 (78.1%)	82 (66.7%)	1	Reference
GA	13 (20.3%)	34 (27.6%)	0.14	0.15 (0.01–1.88)
AA	1 (1.6%)	7 (5.7%)	0.23	0.21 (0.01–2.79)
Alleles *ABCG2* 34G/A	Patients with HIVLD, *N* = 128 (%)	Patients without HIVLD, *N* = 246 (%)	*P*-value	OR (95% CI)
G	113 (88.28%)	198 (80.48%)	1	Reference
A	15 (11.71%)	48 (19.51%)	0.07	0.55 (0.28–1.06)
Genotypes *MTP -*493G/T	Patients with HIVLD, *N* = 64 (%)	Healthy controls, *N* = 139 (%)	*P*-value	OR (95% CI)
GG	28 (43.8%)	61 (43.9%)	1	Reference
GT	26 (40.6%)	50 (36.0%)	0.83	1.13 (0.56–2.29)
TT	10 (15.6%)	28 (20.1%)	0.71	0.78 (0.30–1.96)
Alleles *MTP -*493G/T	Patients with HIVLD, *N* = 128 (%)	Healthy controls, *N* = 278(%)	*P*-value	OR (95% CI)
G	82 (64.06%)	172 (61.87%)	1	Reference
T	46 (35.93%)	106 (38.12%)	0.75	0.91 (0.58–1.44)
Genotypes *ABCG2* 34G/A	Patients with HIVLD, *N* = 64 (%)	Healthy controls, *N* = 139 (%)	*P*-value	OR (95% CI)
GG	50 (78.1%)	91 (65.5%)	1	Reference
GA	13 (20.3%)	44 (31.7%)	0.11	0.54 (0.25–1.15)
AA	1 (1.6%)	4 (2.9%)	0.81	0.45 (0.02–4.51)
Alleles *ABCG2* 34G/A	Patients with HIVLD, *N* = 128 (%)	Healthy controls, *N* = 278 (%)	*P*-value	OR (95% CI)
G	113 (88.28%)	226 (81.29%)	1	Reference
A	15 (11.71%)	52 (18.70%)	0.10	0.58 (0.30–1.11)

HIVLD, HIV-associated lipodystrophy.

*N* = total number of subjects, (%) = frequency of genotypes/alleles, odds ratios (OR), and 95% CI confidence intervals (CI) were derived from logistic regression models comparing the homozygous wild-type genotype/allele (GG genotype and G allele for *MTP* 493G/T polymorphism and GG genotype and G allele for *ABCG2* 34G/A polymorphism were taken as reference) with other genotypes/alleles.

### *MTP* -493G/T and *ABCG2* 34G/A polymorphisms and patients without HIVLD

The genotype and allelic frequencies of *MTP* -493G/T and *ABCG2* 34G**/**A polymorphisms in patients without HIVLD and healthy controls are shown in Table 3. The genotype frequency of *MTP* -493G/T polymorphism did not follow the Hardy–Weinberg equilibrium (*P *= 0.005), while *ABCG2* 34G**/**A polymorphism in healthy controls were in the Hardy–Weinberg equilibrium (*P *= 0.63). The occurrences of *MTP* -493GG, *MTP* -493GT, and *MTP* -493TT and *ABCG*2 34GG, *ABCG*2 34GA, and *ABCG*2 34AA genotypes were almost alike between patients without HIVLD and healthy controls (51.2% vs. 43.9%, 36.6% vs. 36.0%, 12.2% vs. 20.1%, 66.7% vs. 65.5%, 27.6% vs. 31.7%, 5.7% vs. 2.9%). *MTP -*493T allele was distributed almost similarly between patients without HIVLD and healthy controls (37% vs. 38.12%).

**Table 3:** Frequency distribution of *MTP* 493G/T and *ABCG2* 34G/A polymorphisms in between patients without HIVLD and healthy controls.

Genotypes *MTP -* 493G/T	Patients without HIVLD, *N* = 123 (%)	Healthy controls, *N* = 139 (%)	*P*-value	OR (95% CI)
GG	63 (51.2%)	61 (43.9%)	1	Reference
GT	45 (36.6%)	50 (36.0%)	0.71	0.87 (0.49–1.54)
TT	15 (12.2%)	28 (20.1%)	0.10	0.52 (0.24–1.13)
Alleles *MTP -* 493G/T	Patients without, HIVLD *N* = 246 (%)	Healthy controls, *N* = 278 (%)	*P*-value	OR (95% CI)
G	171 (63%)	172 (61.87%)	1	Reference
T	75 (37%)	106 (38.12%)	0.08	0.71 (0.49–1.04)
Genotypes *ABCG2* 34G/A	Patients without HIVLD, *N* = 123 (%)	Healthy controls, *N* = 139 (%)	*P*-value	OR (95% CI)
GG	82 (66.7%)	91 (65.5%)	1	Reference
GA	34 (27.6%)	44 (31.7%)	0.67	0.86 (0.48–1.52)
AA	7 (5.7%)	4 (2.9%)	0.46	1.94 (0.49–8.23)
Alleles *ABCG2* 34G/A	Patients without HIVLD, *N* = 246 (%)	Healthy controls, *N* = 278 (%)	*P*-value	OR (95% CI)
G	198 (80.48%)	226 (81.29%)	1	Reference
A	48 (19.51%)	52 (18.70%)	0.90	1.05 (0.67–1.67)

HIVLD, HIV-associated lipodystrophy.

*N* = total number of subjects, (%) = frequency of genotypes/alleles, odds ratios (OR), and 95% CI confidence intervals (CI) were derived from logistic regression models comparing the homozygous wild-type genotype/allele (GG genotype and G allele for *MTP* 493G/T polymorphism and GG genotype and G allele for *ABCG2* 34G/A polymorphism were taken as reference) with other genotypes/alleles.

### Haplotype analysis of *MTP* -493G/T and *ABCG2* 34G/A polymorphisms

Haplotype frequency of *MTP* -493G/T and *ABCG2* 34G/A polymorphisms in patients with and without HIVLD and healthy controls is shown in Table 4. In the patients with and without HIVLD, patients with HIVLD and healthy controls, and patients without HIVLD and healthy controls populations, there was no significant LD (D’) between both genes (*P* = 0.2247, *P* = 0.225, *P* = 0.2647). Comparing the LD values (Dij) between the patients with HIVLD and without HIVLD, patients with HIVLD and healthy controls, and patients without HIVLD and healthy controls subjects, no significant difference was found between both genes. It was expected that there might be additive or synergistic effect of these variations in modulation of pathogenesis of HIVLD.

**Table 4:** Frequency distribution of haplotypes of *MTP* 493G/T and *ABCG2* 34G/A between patients with and without HIV-associated lipodystrophy and patients with HIV-associated lipodystrophy and healthy controls.

Haplotypes *MTP -*493G/T, *ABCG2* 34G/A	Patients with HIVLD, *N* = 128 (%)	Patients without HIVLD, *N* = 246 (%)	*P*-value	OR (95% CI)
GG	0.567	0.558	1	Reference
TG	0.315	0.266	0.52	1.18 (0.71–1.96)
GA	0.073	0.136	0.17	0.52 (0.20–1.33)
TA	0.044	0.136	0.80	1.19 (0.31–4.62)
Haplotypes *MTP -*493G/T, *ABCG2* 34G/A	Patients with HIVLD, *N* = 246 (%)	Healthy controls, *N* = 278 (%)	*P*-value	OR (95% CI)
GG	0.567	0.473	1	Reference
TG	0.315	0.325	0.75	0.92 (0.54–1.55)
GA	0.073	0.145	0.22	0.55 (0.21–1.42)
TA	0.044	0.056	0.94	0.96 (0.29–3.12)
Haplotypes *MTP -*493G/T, *ABCG2* 34G/A	Patients without HIVLD, *N* = 246 (%)	Healthy controls, *N* = 278 (%)	*P*-value	OR (95% CI)
GG	0.558	0.473	1	Reference
TG	0.266	0.325	0.40	0.82 (0.53–1.28)
GA	0.136	0.145	0.80	0.92 (0.50–1.70)
TA	0.038	0.056	0.83	0.90 (0.34–2.34)

HIVLD, HIV-associated lipodystrophy.

(%) = frequency of subjects, odds ratios, and 95% CIs were derived from logistic regression models comparing the haplotype GG with other haplotypes.

Haplotype GG (*MTP* *G/ABCG2*G) was taken as reference. The frequency of haplotype GG, TG, GA, and TA (*MTP* *G, *T, *G, *T/ABCG2*G, *G, *A, *A) did not differ significantly between patients with and without HIVLD, patients with HIVLD and healthy controls, and patients without HIVLD and healthy controls (56.7% vs. 55.8%, 31.5% vs. 26.6%, 7.3% vs. 13.6%, 4.4% vs. 13.6%, 56.7% vs. 47.3%, 31.5% vs. 32.5%, 7.3% vs. 14.5%, 4.4% vs. 5.6%, 55.8% vs. 47.3%, 26.6% vs. 32.5%, 13.6% vs. 14.5%, 3.8% vs. 5.6%).

### *MTP* -493G/T and *ABCG2* 34G/A polymorphisms and patients with and without HIVLD who have impaired LDL level

The genotype frequencies of *MTP* -493G/T and *ABCG2* 34G/A polymorphisms in patients with and without HIVLD who have impaired LDL level and normal LDL level are shown in Table 5. In patients with HIVLD, *ABCG2* 34GA genotype was distributed significantly lower in normal LDL level compared with impaired LDL level and showed a reduced risk for severity of LDHIV (7.1% vs. 30.6%; *P* = 0.04, OR = 0.17, 95% CI 0.02–0.94). *ABCG2* 34GG genotype was disseminated higher in impaired LDL level than in normal LDL level (92.9% vs. 66.7%). *ABCG2* 34GG, *ABCG2* 34GA, and *ABCG2* 34AA genotypes were dispersed almost alike in impaired LDL level and normal LDL level (64.7% vs. 68.1%, 31.4% vs. 25.0%, 3.9% vs. 6.9%) among patients with HIVLD. In patients with and without HIVLD, *MTP* -493GT and *MTP* -493TT genotypes were disseminated higher in impaired LDL level than in normal LDL level and showed an increased risk for severity of LDHIV and dyslipidemia, respectively (35.7% vs. 50.0%, *P* = 0.43, OR = 1.80, 95% CI 0.53–6.21 and 17.9% vs. 13.9%, *P* = 0.67, OR = 1.80, 95% CI 0.34–9.85 and 39.21% vs. 34.7%, *P* = 34.7%, OR = 1.30, 95% CI 0.56–3.04 and 13.72% vs. 11.1%, *P* = 0.75, OR = 1.42, 95% CI 0.40–5.07), while as *MTP* -493GG genotype was distributed lesser in impaired LDL level than in normal LDL level (47.05% vs. 54.2%).

**Table 5:** Frequency distribution of *MTP* 493G/T and *ABCG2* 34G/A polymorphisms in patients with and without HIVLD who have impaired LDL level.

HIVLD				Without HIVLD			
Genotypes *MTP -*493G/T	Impaired LDL level	Normal LDL level	*P*-value, OR (95% CI)	Genotypes *MTP -*493G/T	Impaired LDL level	Normal LDL level	*P*-value, OR (95% CI), 1 (Reference)
	*N =* 28(%)	*N = *36 (%)			*N =* 51 (%)	*N* *= *72 (%)	
GG	10 (35.7%)	18 (50.0%)	1 (Reference)	GG	24 (47.05%)	39 (54.2%)	1 (Reference)
GT	13 (46.4%)	13 (36.1%)	0.43, 1.80 (0.53–6.21)	GT	20 (39.21%)	25 (34.7%)	0.64, 1.30 (0.56–3.04)
TT	5 (17.9%)	5 (13.9%)	0.67, 1.80 (0.34–9.85)	TT	7 (13.72%)	8 (11.1%)	0.75, 1.42 (0.40–5.07)
Genotypes *ABCG2* 34G/A	Impaired LDL level	Normal LDL level	*P*-value OR (95% CI)	Genotypes *ABCG2* 34G/A	Impaired LDL level	Normal LDL level	*P*-value OR (95% CI)
	*N =* 28(%)	*N = *36 (%)			*N =* 51 (%)	*N = *72 (%)	
GG	26 (92.9%)	24 (66.7%)	1 (Reference)	GG	33 (64.7%)	49 (68.1%)	1 (Reference)
GA	2 (7.1%)	11 (30.6%)	**0.04, 0.17** (**0.02–0.94)**	GA	16 (31.4%)	18 (25.0%)	0.63, 1.32 (0.55–3.19)
AA	0 (0.0%)	1 (2.8%)	—	AA	2 (3.9%)	5 (6.9%)	0.83, 0.59 (0.07–3.78)

HIVLD, HIV-associated lipodystrophy; LDL, low-density lipoprotein.

*N* = total number of subjects, (%) = frequency of genotypes/alleles, odds ratios (OR), and 95% CI confidence intervals (CI) were derived from logistic regression models comparing the homozygous wild-type genotype/allele (GG genotype for *MTP -*493G/T polymorphism and GG genotype for *ABCG2* 34G/A polymorphism were taken as reference) with other genotypes. Significant values (<0.05) represented in bold.

### *MTP* -493G/T and *ABCG2* 34G/A polymorphisms and patients with and without HIVLD who have impaired HDL level

The genotype frequencies of *MTP* -493G/T and *ABCG2* 34G/A polymorphisms in patients with and without HIVLD who have impaired HDL level and normal HDL level are shown in Table 6. In patients with and without HIVLD, *MTP* -493TT genotype displayed an increased risk for severity of LDHIV and dyslipidemia, respectively (37.5% vs. 12.5%, *P* = 0.19, OR = 5.57, 95% CI 0.58–62.28 and 20.0% vs. 11.1%, *P* = 0.62, OR = 2.00, 95% CI 0.35–10.61) when compared between impaired HDL level and normal HDL level, while the occurrences of *MTP* -493GG and *MTP* -493TT genotypes were disseminated lesser in impaired HDL level than in normal HDL level (25.0% vs. 46.4% and 37.5% vs. 41.1%; 46.66% vs. 51.9% and 33.33% vs. 37.0%). In patients with HIVLD, *ABCG2* 34GG genotype distributed lesser in impaired HDL level, while *ABCG2* 34GA genotype disseminated higher in impaired HDL level than in normal HDL level (75.0% vs. 78.6%, 25.0% vs. 19.6%). *ABCG2* 34GG genotype was distributed greater in impaired HDL level, while *ABCG2* 34GA genotype was disseminated lesser in impaired HDL level than in normal HDL level (86.7% vs. 63.9%, 13.3% vs. 29.6%) among patients without HIVLD.

**Table 6:** Frequency distribution of *MTP -*493G/T and *ABCG2* 34G/A polymorphisms in patients with HIVLD and patients without HIVLD who have impaired HDL level.

HIVLD				Without HIVLD			
Genotypes *MTP -*493G/T	Impaired HDL level	Normal HDL level	*P*-value, OR (95% CI)	Genotypes *MTP -*493G/T	Impaired HDL level	Normal HDL level	*P*-value, OR (95% CI), 1 (reference)
	*N = *8 (%)	*N = 5*6 (%)			*N =* 15 (%)	*N* *= *108(%)	
GG	2 (25.0%)	26 (46.4%	1 (reference)	GG	7 (46.66%)	56 (51.9%)	1 (reference)
GT	3 (37.5%)	23 (41.1%)	0.40, 0.30 (0.04–2.51)	GT	5 (33.33%)	40 (37.0%)	0.75, 1.00 (0.25–3.86)
TT	3 (37.5%)	7 (12.5%)	0.19, 5.57 (0.58–62.28)	TT	3 (20.0%)	12 (11.1%)	0.62, 2.00 (0.35–10.61)
Genotypes *ABCG2* 34G/A	Impaired HDL level	Normal HDL level	*P*-value OR (95% CI)	Genotypes *ABCG2* 34G/A	Impaired HDL level	Normal HDL level	*P*-value OR (95% CI)
	*N = *8 (%)	*N = 5*6 (%)			*N =* 15 (%)	*N* *= *108(%)	
GG	6 (75.0%)	44 (78.6%)	1 (reference)	GG	13 (86.7%)	69 (63.9%)	1 (reference)
GA	2 (25.0%)	11 (19.6%)	0.88, 1.33 (0.16–9.11)	GA	2 (13.3%)	32 (29.6%)	0.24, 0.33 (0.05–1.69)
AA	0 (0.0%)	1 (1.8%	—	AA	0 (0.0%)	7 (6.5%)	—

HIVLD, HIV-associated lipodystrophy.

*N* = total number of subjects, (%) = frequency of genotypes/alleles, odds ratios (OR), and 95% CI confidence intervals (CI) were derived from logistic regression models comparing the homozygous wild-type genotype/allele (GG genotype for *MTP -*493G/T polymorphism and GG genotype for *ABCG2* 34G/A polymorphism were taken as reference) with other genotypes.

### *MTP* -493G/T and *ABCG2* 34G/A polymorphisms and patients with and without HIVLD who have impaired triglyceride level

The genotype frequencies of *MTP* -493G/T and *ABCG2* 34G/A polymorphisms in patients with and without HIVLD who have impaired triglyceride level and normal triglyceride level are shown in Table 7. *MTP* -493GT and *MTP* -493TT genotypes exhibited a risk for severity of LDHIV and dyslipidemia, respectively (50.0% vs. 39.3%, *P* = 0.59, OR = 2.36, 95% CI 0.32–20.8 and 25.0% vs. 14.3%, *P* = 0.59, OR = 3.25, 95% CI 0.270–40.83; 40.0% vs. 36.3%, *P* = 0.64, OR = 1.95, 95% CI 0.34–11.73 and 30.0% vs.10.6%, *P* = 0.14, OR = 5.00, 95% CI 0.69–36.83) when compared between impaired triglyceride level and normal triglyceride level among patients with and without HIVLD. In patients with and without HIVLD, *ABCG2* 34GG genotype distribution was dissimilar in impaired triglyceride level than in normal triglyceride level (87.5% vs. 76.8% and 70.0% vs. 66.4%). In patients without HIVLD, *ABCG2* 34GA genotype showed an increased risk for dyslipidemia with borderline significance when compared between impaired triglyceride level and normal triglyceride level (53.33% vs. 27.4%, *P* = 0.07, OR = 2.76, 95% CI 0.82–9.43). *ABCG2* 34GA genotype was distributed differently between impaired triglyceride level and normal triglyceride level (12.5% vs. 21.4%) among patients with HIVLD.

**Table 7:** Frequency distribution of *MTP* 493G/T and *ABCG2* 34G/A polymorphisms in patients with HIVLD and patients without HIVLD who have impaired triglyceride level.

HIVLD				Without HIVLD			
Genotypes *MTP -*493G/T	Impaired triglyceride level	Normal triglyceride level	*P*-value, OR (95% CI)	Genotypes *MTP -*493G/T	Impaired triglyceride level	Normal triglyceride level	*P*-value, OR (95% CI), 1 (reference)
	*N =* 8 (%)	*N = *56 (%)			*N =* 10 (%)	*N* *= *113(%)	
GG	2 (25.0%)	26 (46.4%)	1 (reference)	GG	3 (30.0%)	60 (53.1%)	1 (reference)
GT	4 (50.0%)	22 (39.3%)	0.59, 2.36 (0.32–20.84)	GT	4 (40.0%)	41 (36.3%)	0.64, 1.95 (0.34–11.73)
TT	2 (25.0%)	8 (14.3%)	0.59, 3.25 (0.270–40.83)	TT	3 (30.0%)	12 (10.6%)	0.14, 5.00 (0.69–36.83)
Genotypes *ABCG2* 34G/A	Impaired triglyceride level	Normal triglyceride level	*P*-value, OR (95% CI)	Genotypes *ABCG2* 34G/A	Impaired triglyceride level	Normal triglyceride level	*P*-value, OR (95% CI)
	*N = *8 (%)	*N = 5*6 (%)			*N =* 15 (%)	*N* *= *108 (%)	
GG	7 (87.5%)	43 (76.8%)	1 (reference)	GG	7 (46.66%)	75 (66.4%)	1 (reference)
GA	1 (12.5%)	12 (21.4%)	0.88, 0.51 (0.02–5.00)	GA	8 (53.33%)	31 (27.4%)	0.07, 2.76 (0.82–9.43)
AA	0 (0.0%)	1 (1.8%)	—	AA	0 (0.0%)	7 (6.2%)	—

HIVLD, HIV-associated lipodystrophy.

*N* = total number of subjects, (%) = frequency of genotypes/alleles, odds ratios (OR), and 95% CI confidence intervals (CI) were derived from logistic regression models comparing the homozygous wild-type genotype/allele (GG genotype for *MTP -*493G/T polymorphism and GG genotype for *ABCG2* 34G/A polymorphism were taken as reference) with other genotypes.

### *MTP* -493G/T and *ABCG2* 34G/A polymorphisms and patients with and without HIVLD who have impaired cholesterol level

The genotype frequencies of *MTP* -493G/T and *ABCG2* 34G/A polymorphisms in patients with and without HIVLD who have impaired cholesterol level and normal cholesterol level are shown in Table 8. In patients with HIVLD, *MTP* -493GT and *MTP* -493TT genotypes showed a risk for severity of LDHIV (66.7% vs. 37.9%, *P* = 0.30, OR = 4.91, 95% CI 0.45–124.25 and 16.7% vs. 15.5%, *P* = 0.96, OR = 3.00, 95% CI 0.0–125.67) when compared between impaired cholesterol level and normal cholesterol level. *MTP* -493GT and *MTP* -493GG genotypes was distributed lesser in impaired cholesterol level than normal cholesterol level (16.7% vs. 46.6%). In patients without HIVLD, *ABCG2* 34AA genotype displayed a risk for dyslipidemia (13.3% vs. 13.3%, *P* = 0.44, OR = 3.24, 95% CI 0.37–23.98), and *ABCG2* 34GG and *ABCG2* 34GA genotypes were distributed almost similarly (60.0% vs. 67.6%; 26.7% vs. 27.8%) when compared between impaired cholesterol level and normal cholesterol level. *MTP* -493GG genotype was dispersed higher, and *MTP* -493GT genotype was dispersed lesser in impaired cholesterol level than in normal cholesterol level (73.3% vs. 48.1%; 26.7% vs. 38.0%).

**Table 8:** Frequency distribution of *MTP -*493G/T and *ABCG2* 34G/A polymorphisms in patients with HIVLD and patients without HIVLD who have impaired total cholesterol level.

HIVLD				Without HIVLD			
Genotypes *MTP -*493G/T	Impaired cholesterol level	Normal cholesterol level	*P*-value, OR (95% CI)	Genotypes *MTP -*493G/T	Impaired cholesterol level	Normal cholesterol level	*P*-value, OR (95% CI), 1 (reference)
	*N =* 6 (%)	*N = *58 (%)			*N =* 15 (%)	*N* *= *108(%)	
GG	1 (16.7%)	27 (46.6%)	1 (reference)	GG	11 (73.3%)	52 (48.1%)	1 (reference)
GT	4 (66.7%)	22 (37.9%)	0.30, 4.91 (0.45–124.25)	GT	4 (26.7%)	41 (38.0%)	0.32, 0.46 (0.11–1.73)
TT	1 (16.7%)	9 (15.5%)	0.96, 3.00 (0.0–125.67)	TT	0 (0.0%)	15 (13.9%)	—
Genotypes *ABCG2* 34G/A	Impaired cholesterol level	Normal cholesterol level	*P*-value OR (95% CI)	Genotypes *ABCG2* 34G/A	Impaired cholesterol level	Normal cholesterol level	*P*-value OR (95% CI)
	*N = *6 (%)	*N = 5*8 (%)			*N =* 10 (%)	*N* *= *113 (%)	
GG	6 (100.0%)	44 (75.9%)	1 (reference)	GG	9 (60.0%)	73 (67.6%)	1 (reference)
GA	0 (0.0%)	13 (22.4%)	—	GA	4 (26.7%)	30 (27.8%)	0.84, 1.08 (0.26–4.27)
AA	0 (0.0%)	1 (22.4%)	—	AA	2 (13.3%)	5 (4.6%)	0.44, 3.24 (0.37–23.98)

HIVLD, HIV-associated lipodystrophy.

*N* = total number of subjects, (%) = frequency of genotypes/alleles, odds ratios (OR), and 95% CI confidence intervals (CI) were derived from logistic regression models comparing the homozygous wild-type genotype/allele (GG genotype for *MTP* 493G/T polymorphism and GG genotype for *ABCG2* 34G/A polymorphism were taken as reference) with other genotypes.

**Table 9:** Frequency distribution of *MTP* 493G/T and *ABCG2* 34G/A polymorphisms in patients with and without HIVLD who have impaired fasting glucose level.

HIVLD						Without HIVLD					
Genotypes *MTP -*493G/T	Normal level	Prediabetic	*P*-value, OR (95% CI)	Impaired fasting glucose/diabetic	*P*-value, OR (95% CI)	Genotypes *MTP -*493G/T	Normal	Prediabetic	*P*-value, OR (95% CI), 1 (Reference)	Impaired fasting glucose	*P*-value, OR (95% CI), 1 (Reference)
	*N = *57 (%)	*N* = 5(%)		*N =* 2 (%)			*N* *= *99 (%)	*N* = 13 (%)		*N =* 11 (%)	
GG	26 (45.6%)	1 (20.0%)	1 (Reference)	1 (50.0%)	1 (Reference)	GG	50 (50.5%)	8 (61.5%)	1 (Reference)	5 (45.5%)	1 (Reference)
GT	22 (38.6%)	3 (60.0%)	0.54, 3.55 (0.29–95.37)	1 (50.0%)	0.54, 1.18 (0.0–46.51)	GT	38 (38.4%)	4 (30.8%)	0.73,0.66 (0.15–2.67)	3 (27.3%)	0.95, 0.79 (0.14–4.13)
TT	9 (15.8%)	1 (20.0%)	0.94, 2.89 (0.0–121.17)	0 (0.0%)	—	TT	11 (11.1%)	1 (7.7%)	0.96,0.57 (0.02–5.44)	3 (27.3%)	0.41, 2.73 (0.43–16.34)
Genotypes *ABCG2* 34G/A	Normal	Prediabetic	*P*-value, OR (95% CI)	Impaired fasting glucose/diabetic	*P*-value, OR (95% CI)	Genotypes *ABCG2* 34G/A	Normal			Impaired fasting glucose	*P*-value, OR (95% CI)
	*N = 5*7 (%)	*N* = 5		*N = *2 (%)			*N = *99 (%)	*N* = 13 (%)		*N =* 11 (%)	
GG	44 (77.2%)	5 (100.0%)	Reference)	1 (50.0%)	1 (Reference)	GG	66 (66.7%)	8 (61.5%)	1 (Reference)	8 (72.7%)	1 (Reference)
GA	12 (21.1%)	—	—	1 (50.0%)	0.92–3.67 (0.0–147.57)	GA	27 (27.3%)	4 (30.8%)	0.97,1.22 (0.28–5.02)	3 (72.7%)	0.81, 0.92 (0.18–4.23)
AA	1 (1.8%)	—	—	—	—	AA	6 (6.1%)	1 (7.7%)	0.72, 1.38 (cornfield limits invalid	0.0	—

HIVLD, HIV-associated lipodystrophy.

*N* = total number of subjects, (%) = frequency of genotypes/alleles, odds ratios (OR), and 95% CI confidence intervals (CI) were derived from logistic regression models comparing the homozygous wild-type genotype/allele (GG genotype for *MTP* 493G/T polymorphism and GG genotype for *ABCG2* 34G/A polymorphism were taken as reference) with other genotypes.

### *MTP -*493G/T and *ABCG2* 34G/A polymorphisms and patients with and without HIVLD who have impaired fasting glucose level

The genotype frequencies of *MTP -*493G/T and *ABCG2* 34G/A polymorphisms in patients with and without HIVLD who have impaired fasting glucose level/diabetic, are prediabetic, and have normal glucose level are presented in Table 8. In patient with HIVLD, the occurrence of *MTP* 493GT genotypes was dissimilar between prediabetic and normal glucose level (60.0% vs. 38.6%, P = 0.54, OR = 3.55, 95% CI 0.29–95.37) and showed an insignificant risk for severity of HIVLD. In patient with HIVLD, the occurrence of *ABCG*2 34GA genotypes was different between diabetic and normal glucose level (50.0% vs. 21.1%, *P* = 0.92, OR = 3.67, 95% CI 0.0–147.57) and showed an insignificant risk for severity of HIVLD.

In patient without HIVLD, the occurrence of *MTP* 493GT genotypes was almost similar among diabetic, prediabetic, and normal glucose level (38.4% vs. 30.8% vs. 27.3%). In patients without HIVLD, the occurrence of *MTP* -493TT genotype was not significantly higher in diabetic glucose level than in normal glucose level and showed a risk for development of dyslipidemia (27.3 vs. 11.1%; *P* = 0.41, OR = 2.73, 95% CI 0.43–16.34). In patients without HIVLD, the occurrences of *ABCG2* 34GG and *ABCG2* 34GA genotypes were almost similar among diabetic, prediabetic, and normal glucose level (72.72% vs. 61.5% vs. 66.7% and 27.27% vs. 30.8% vs. 27.3%).

### Expression level of *MTP* and *ABCG2* genes

**Figure 1:**
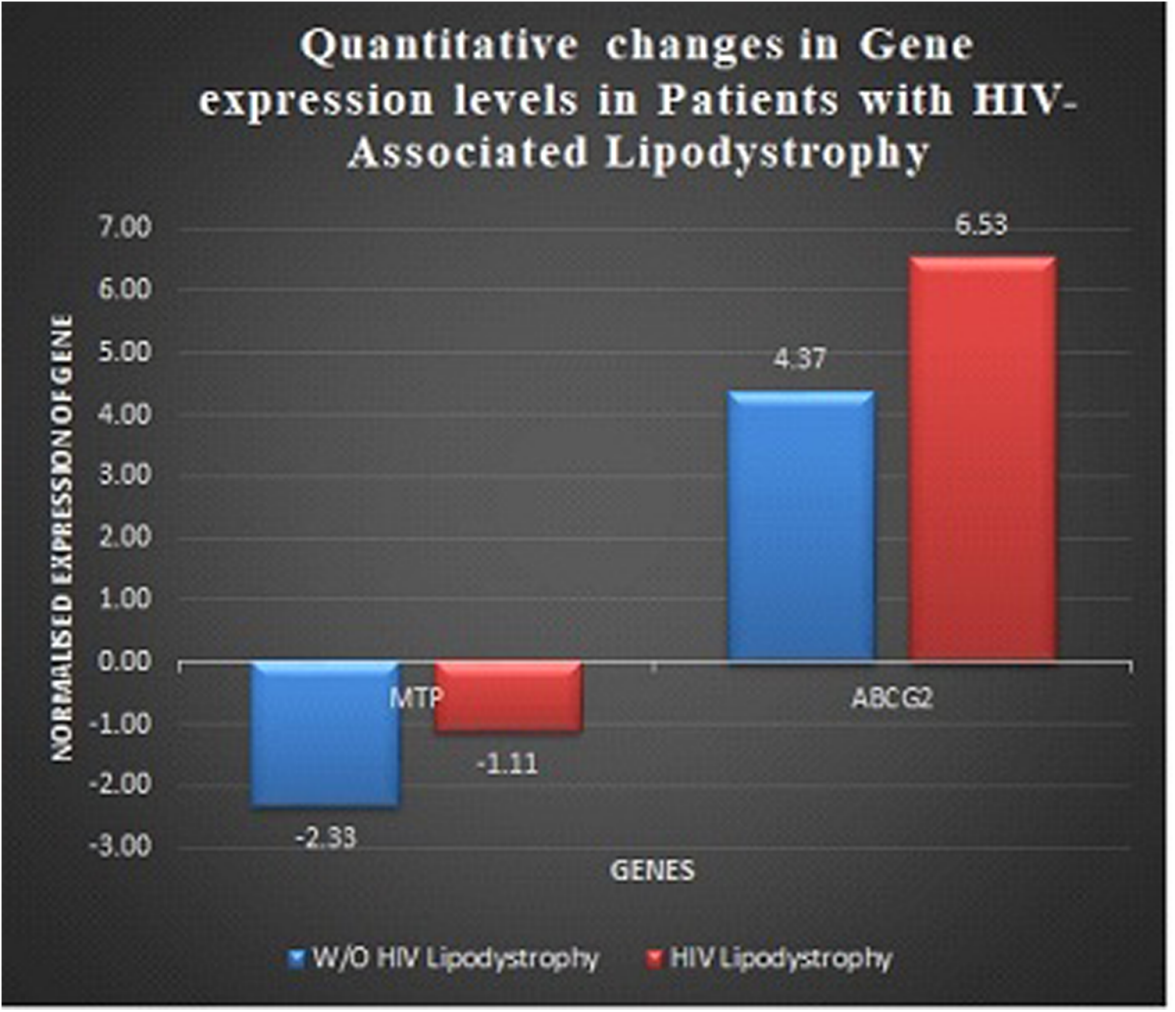
Quantitative changes in gene expression levels in patients with HIV-associated lipodystrophy. Quantitative changes in gene expression levels of *MTP* and *ABCG2* genes in patients with and without HIV-associated lipodystrophy.

The expression analysis of *MTP* and *ABCG2* genes in a total of 72 HIV-infected individuals (24 patients with HIVLD and 48 patients without HIVLD) is shown in Figure 1. The *BAC2* gene was used as an internal control target. The *MTP* gene was downregulated 1.21-fold higher in patients without HIVLD than in patients with HIVLD (−1.11 vs. −2.33; 1.21-fold). *ABCG2* gene was upregulated 2.16-fold higher in patients with HIVLD than in patients without HIVLD (+6.53 vs. +4.37; 2.16-fold).

## Discussion

This is the first study which independently evaluates the influence of the *MTP -*493G/T and *ABCG2* 34G/A polymorphisms and its haplotype with respect to the risk of dyslipidemia and severity of LDHIV from Western India. Metabolic complications of ART including LDHIV have grown to be a significant issue for the long-term, effective management of HIV infection. The effects of HIV and antiretroviral therapies on fat vary between individuals. Genetic constitution of any individual plays a significant role in inter-patient variability of fat transport, absorption, and elimination. The gene involved in lipid transport plays an important role to modulate plasma TG and HDL-C levels in HIV-infected patients (11). SNPs in genes having a role in lipid transport, absorption, and elimination may help understand individual differences in morphologic and metabolic abnormalities which may develop during HAART treatment (32).

*MTP* and *ABCG2* play an important role in the transport of lipoproteins. *MTP* is necessary for the cellular synthesis and release of lipoproteins containing apolipoprotein B (ApoB) (15, 16). The crucial factor controlling lipoprotein secretion is the concentration of *MTP* in the ER (36). The plasma levels of VLDL and LDL are predicted to be influenced by the secretory pattern of ApoB-containing lipoproteins. Genetic variations can alter the amount or activity of *MTP* in the ER, as well as the lipoprotein secretory pattern and the function of *MTP* (17). *ABCG2* is involved in the transport of bile and lipoprotein (25). The expression pattern and transporter activity of *ABCG2* protein are influenced by the *ABCG2* 34G/A polymorphism.

In the present study, the prevalence of the *MTP -*493G/T polymorphism in healthy subjects was comparable to that in the studies of Gouda et al. (23) and incomparable to the studies of Oliveira et al. (37) and Ledmyr et al. (38). *MTP* -493T allele disclosed a non-significantly reduced dyslipidemia risk (*P* = 0.08, OR = 0.71) when compared between patients without HIVLD and healthy controls. *MTP* -493G/T polymorphism was anticipated to play a significant role in the development of dyslipidemia in type 2 diabetics (39). The occurrence of *MTP* -493GG genotype was greater in non-alcoholic steatohepatitis (NASH) patients compared with that in the controls (*P* = 0.002, 95% CI 2.9–392) (40). *MTP* -493G allele was slightly different between biopsy-proven NASH and simple steatosis (37).

In our study, the frequency of *ABCG2* 34G/A polymorphism in healthy individuals was different as compared with research done by Wang et al. (41), Wu et al. (35)**,** and Sari et al. (42). *ABCG2* 34A allele showed a reduced risk for severity of LDHIV with borderline significance (*P* = 0.07, OR = 0.55). *ABCG2* 34AA genotype displayed a non-significant risk for development of dyslipidemia (*P* = 0.46, OR = 1.94). *ABCG2* 34GA and *ABCG2* 34AA genotypes were substantially linked to an increased risk of breast cancer (35). The *ABCG2* 34G/A polymorphism was frequently observed in a Korean population (43).

Our study has also sought to evaluate the significance of two important genes (*MTP* and *ABCG2* genes) cluster both individually and collectively that are physically connected to each other in patients with and without HIVLD. Haplotype analysis, which evaluates interactions between two or more genes that are near to each other, has an advantage over a single-locus analysis in terms of disease susceptibility. In our study, none of haplotype was associated with the higher risk for severity of LDHIV and dyslipidemia.

In our study, *ABCG2* 34GA genotype was linked with the reduced risk for severity of LDHIV (*P* = 0.04, OR = 0.17), whereas *MTP* -493GT and *MTP* -493TT genotypes showed an insignificant risk for severity of LDHIV (*P* = 0.43, OR = 1.80; *P* = 0.67, OR = 1.80) when compared between impaired LDL level and normal LDL level. This suggests that patients with HIVLD who have impaired LDL level and *MTP* -493GT and *MTP* -493TT genotypes may have a risk for severity of LDHIV. It has been demonstrated that *MTP* -493G/T polymorphism affects transcriptional activity and is linked to low plasma levels of LDL cholesterol in healthy middle-aged males (14). In individuals with familial hypercholesterolemia (FH), the *MTP*-493T variant allele's LDL cholesterol-lowering function is switched to a triglyceride-lowering effect (44). Changes in VLDL and LDL were linked to *MTP* -493G/T polymorphism in individuals with type 2 diabetes (45).

In our study, *MTP -*493TT genotype displayed a non-significant risk for severity of LDHIV and dyslipidemia, respectively (*P* = 0.19, OR = 5.57; *P* = 0.62, OR = 2.00) when compared between impaired HDL level and normal HDL level among patients with and without HIVLD. This suggests that patients with and without HIVLD who have impaired HDL level and *MTP-*493TT genotype may have a risk for severity of LDHIV and dyslipidemia, respectively.

In the present study, *MTP* -493GT and *MTP* -493TT genotypes demonstrated an insignificant increased risk for severity of LDHIV and dyslipidemia, respectively (*P* = 0.59, OR = 2.36; *P* = 0.59, OR = 3.25 and *P* = 0.64, OR = 1.95; *P* = 0.14, OR = 5.00) when compared between impaired triglyceride level and normal triglyceride level among patients with and without HIVLD. *ABCG2* 34GA genotype was likely to be associated with impaired triglyceride level with marginal significance and have been shown an increased risk for dyslipidemia (*P* = 0.07, OR = 2.76) when compared between impaired triglyceride level and normal triglyceride level among patients without HIVLD. This suggests that patients with and without HIVLD who have impaired HDL level and *MTP* -493GT and *MTP* -493TT and *ABCG2* 34GA genotypes may have a risk for severity of LDHIV and dyslipidemia, respectively. The *ABCG2* 34G/A polymorphism affects the *ABCG2* expression pattern and its transporter activity (31, 32). Fewer but more lipid-rich VLDL particles were found in the *MTP* -493TT variant genotype, corroborating the notion that *MTP* expression affects the hepatic secretion of triglyceride-rich, ApoB-containing lipoproteins (14).

*MTP -*493GT and *MTP* -493TT genotypes exhibited a non-significant risk for severity of LDHIV (*P* = 0.30, OR = 4.91; *P* = 0.96, OR = 3.00) when compared between impaired cholesterol level and normal cholesterol level among patients with HIVLD. *ABCG2* 234AA genotype exposed an insignificant risk for development of dyslipidemia (*P* = 0.44, OR = 3.24) when compared between impaired cholesterol level and normal cholesterol level among patients without HIVLD. This suggests that patients with and without HIVLD who have impaired cholesterol level and *MTP* -493GT and *MTP* -493TT and *ABCG2* 34AA genotypes may have a risk for severity of LDHIV and dyslipidemia, respectively.

In patients without HIVLD, individuals with *MTP -*493TT genotype have shown a risk for development of HIVLD (*P* = 0.41, OR = 2.73) when compared between diabetic and normal glucose level. However, risk could not reach statistical significance. This suggests that patients without HIVLD who have impaired fasting glucose level and *MTP* -493TT genotypes may have a risk for dyslipidemia. *MTP* -493G/T polymorphism was associated with gene expression and resulted in aberrant alterations of MTP synthesis and secretion, affecting the capacity for lipid export, which induces dysregulation of hepatic lipid metabolism (22, 23).

### Effect of *MTP* -493G/T and *ABCG2* 34G/A polymorphisms on expression level

In the present study, *MTP* gene was downregulated 1.21-fold in patients without HIVLD because *MTP* -493T allele was expressed lesser in patients without HIVLD compared with that in patients with HIVLD and healthy subjects (*P* = 0.34, OR = 1.28; *P *=*** ***0.08, OR = 0.71) and correlated with the decreased *MTP* expression. *MTP* -493G/T polymorphism was linked to altered gene expression and MTP secretion, which affected the ability to transport lipids (22, 23).

In our study, *ABCG2* gene was upregulated 2.16-fold in patients with HIVLD. Reduced production of the *ABCG2* protein and subsequently reduced transporter activity are linked to the *ABCG2* 34G/A polymorphism (30, 32–34). The overexpression of *ABCG2* has been reported to lead to inter-individual difference in bioavailability of drugs (26).

### Limitations

This study has certain limitations. It could only evaluate the association and not decide causality. Initially, we allocated a ratio of 1:4 for case-controls. However, we were unable to manage matched enrollment in the controls. Despite this, our case-to-control ratio is approximately 1:2, which may be sufficient for this study. In addition, we had not assessed the plasma drug concentration in our subject participants. Hence, we were not able to address the correlation of *MTP* and *ABCG2* polymorphisms with plasma drug levels in HIV patients.

## Conclusion

The expression level of MTP gene was influenced by the *MTP* -493C/T polymorphism. Individuals who have impaired triglyceride level with *ABCG2* 34GA genotype may have a risk of dyslipidemia. Individuals with independently *ABCG2* 34A allele and *ABCG2* 34GA genotype with impaired LDL level may have a role to reduce the risk for severity of LDHIV. Similarly, *MTP* -493T allele may also reduce the risk for dyslipidemia. Further studies should be carried out in same and other populations with larger sample size for better understanding of the role of transporters among LDHIV.

## Data Availability

The original contributions presented in the study are included in the article/Supplementary Materials, and further inquiries can be directed to the corresponding author.

## References

[1] Loonam CR Mullen A. Nutrition and the HIV-associated lipodystrophy syndrome. Nutr Res Rev. (2012) 25(2):267–87. 10.1017/S0954422411000138 23174511

[2] Miller J Carr A Emery S Law M Mallal S Baker D HIV lipodystrophy: prevalence, severity and correlates of risk in Australia. HIV Med. (2003) 4(3):293–301. 10.1046/j.1468-1293.2003.00159.x 12859330

[3] Jacobson DL Knox T Spiegelman D Skinner S Gorbach S Wanke C. Prevalence of, evolution of, and risk factors for fat atrophy and fat deposition in a cohort of HIV-infected men and women. Clin Infect Dis. (2005) 40(12):1837–45. 10.1086/430379 15909274

[4] Hansen AB Lindegaard B Obel N Andersen O Nielsen H Gerstoft J. Pronounced lipoatrophy in HIV-infected men receiving HAART for more than 6 years compared with the background population. HIV Med. (2006) 7(1):38–45. 10.1111/j.1468-1293.2005.00334.x 16313291

[5] Mercier S Gueye NF Cournil A Fontbonne A Copin N Ndiaye I Lipodystrophy and metabolic disorders in HIV-1-infected adults on 4- to 9-year antiretroviral therapy in Senegal: a case-control study. J Acquir Immune Defic Syndr. (2009) 51(2):224–30. 10.1097/QAI.0b013e31819c16f4 19339897

[6] Kalyanasundaram AP Jacob SM Hemalatha R Sivakumar MR. Prevalence of lipodystrophy and dyslipidemia among patients with HIV infection on generic ART in rural south India. J Int Assoc Physicians AIDS Care. (2012) 11(5):329–34. 10.1177/1545109711401750 21508297

[7] Bhutia E Hemal A Yadav TP Ramesh KL. Lipodystrophy syndrome among HIV infected children on highly active antiretroviral therapy in northern India. Afr Health Sci. (2014) 14(2):408–13. 10.4314/ahs.v14i2.17 25320591 PMC4196396

[8] Diehl LA Dias JR Paes AC Thomazini MC Garcia LR Cinagawa E Prevalência da lipodistrofia associada ao HIV em pacientes ambulatoriais brasileiros: relação com síndrome metabólica e fatores de risco cardiovascular [Prevalence of HIV-associated lipodystrophy in Brazilian outpatients: relation with metabolic syndrome and cardiovascular risk factors]. Arq Bras Endocrinol Metabol. (2008) 52(4):658–67 (in Portuguese). 10.1590/S0004-27302008000400012 18604379

[9] Lichtenstein KA Ward DJ Moorman AC Delaney KM Young B Palella FJ Jr. Clinical assessment of HIV-associated lipodystrophy in an ambulatory population. AIDS. (2001) 15(11):1389–98. 10.1097/00002030-200107270-00008 11504960

[10] Alencastro PR Barcellos NT Wolff FH Ikeda ML Schuelter-Trevisol F Brandão AB People living with HIV on ART have accurate perception of lipodystrophy signs: a cross-sectional study. BMC Res Notes. (2017) 10(1):40. 10.1186/s13104-017-2377-3 28086977 PMC5234247

[11] Tarr PE Telenti A. Toxicogenetics of antiretroviral therapy: genetic factors that contribute to metabolic complications. Antivir Ther. (2007) 12(7):999–1013. 10.1177/135965350701200714 18018758

[12] Knox TA Wanke C. 397 – gastrointestinal manifestations of HIV and AIDS. In: Goldman L, Schafer AI, editors. Goldman’s Cecil medicine (twenty-fourth edition). W.B. Saunders (2012). p. 2196–9. 10.1016/B978-1-4377-1604-7.00397-3

[13] Hussain MM Rava P Walsh M Rana M Iqbal J. Multiple functions of microsomal triglyceride transfer protein. Nutr Metab. (2012) 9:14. 10.1186/1743-7075-9-14 PMC3337244 22353470

[14] Karpe F Lundahl B Ehrenborg E Eriksson P Hamsten A. A common functional polymorphism in the promoter region of the microsomal triglyceride transfer protein gene influences plasma LDL levels. Arterioscler Thromb Vasc Biol. (1998) 18(5):756–61. 10.1161/01.ATV.18.5.756 9598834

[15] Iqbal J Rudel LL Hussain MM. Microsomal triglyceride transfer protein enhances cellular cholesteryl esterification by relieving product inhibition. J Biol Chem. (2008) 283(29):19967–80. 10.1074/jbc.M800398200 18502767 PMC2459284

[16] Wetterau JR Combs KA Spinner SN Joiner BJ. Protein disulfide isomerase is a component of the microsomal triglyceride transfer protein complex. J Biol Chem. (1990) 265(17):9800–7. 10.1016/S0021-9258(19)38742-3 2351674

[17] Mirandola S Bowman D Hussain MM Alberti A. Hepatic steatosis in hepatitis C is a storage disease due to HCV interaction with microsomal triglyceride transfer protein (MTP). Nutr Metab. (2010) 7:13. 10.1186/1743-7075-7-13 PMC2838899 20178560

[18] Mirandola S Osterreicher CH Marcolongo M Datz C Aigner E Schlabrakowski A Microsomal triglyceride transfer protein polymorphism (-493G/T) is associated with hepatic steatosis in patients with chronic hepatitis C. Liver Int. (2009) 29(4):557–65. 10.1111/j.1478-3231.2008.01892.x 19018985

[19] Siqueira ER Oliveira CP Correa-Giannella ML Stefano JT Cavaleiro AM Fortes MA MTP -493G/T gene polymorphism is associated with steatosis in hepatitis C-infected patients. Braz J Med Biol Res. (2012) 45(1):72–7. 10.1590/S0100-879X2011007500160 22147193 PMC3854139

[20] Bernard S Touzet S Personne I Lapras V Bondon PJ Berthezène F Association between microsomal triglyceride transfer protein gene polymorphism and the biological features of liver steatosis in patients with type II diabetes. Diabetologia. (2000) 43(8):995–9. 10.1007/s001250051481 10990076

[21] Namikawa C Shu-Ping Z Vyselaar JR Nozaki Y Nemoto Y Ono M Polymorphisms of microsomal triglyceride transfer protein gene and manganese superoxide dismutase gene in non-alcoholic steatohepatitis. J Hepatol. (2004) 40(5):781–6. 10.1016/j.jhep.2004.01.028 15094225

[22] Dowman JK Tomlinson JW Newsome PN. Pathogenesis of non-alcoholic fatty liver disease. QJM. (2010) 103(2):71–83. 10.1093/qjmed/hcp158 19914930 PMC2810391

[23] Gouda W Ashour E Shaker Y Ezzat W. MTP genetic variants associated with non-alcoholic fatty liver in metabolic syndrome patients. Genes Dis. (2017) 4(4):222–8. 10.1016/j.gendis.2017.09.002 30258926 PMC6147179

[24] Ankathil R Azlan H Dzarr AA Baba AA. Pharmacogenetics and the treatment of chronic myeloid leukemia: how relevant clinically? An update. Pharmacogenomics. (2018) 19(5):475–393. 10.2217/pgs-2017-0193 29569526

[25] Robey RW To KK Polgar O Dohse M Fetsch P Dean M ABCG2: a perspective. Adv Drug Deliv Rev. (2009) 61(1):3–13. 10.1016/j.addr.2008.11.003 19135109 PMC3105088

[26] Loscocco F Visani G Galimberti S Curti A Isidori A. BCR-ABL independent mechanisms of resistance in chronic myeloid leukemia. Front Oncol. (2019) 9:939. 10.3389/fonc.2019.00939 31612105 PMC6769066

[27] Eyal S Hsiao P Unadkat JD. Drug interactions at the blood-brain barrier: fact or fantasy? Pharmacol Ther. (2009) 123(1):80–104. 10.1016/j.pharmthera.2009.03.017 19393264 PMC2751796

[28] Vasiliou V Vasiliou K Nebert DW. Human ATP-binding cassette (ABC) transporter family. Hum Genomics. (2009) 3(3):281–90. 10.1186/1479-7364-3-3-281 19403462 PMC2752038

[29] Bäckström G Taipalensuu J Melhus H Brändström H Svensson AC Artursson P Genetic variation in the ATP-binding cassette transporter gene ABCG2 (BCRP) in a Swedish population. Eur J Pharm Sci. (2003) 18(5):359–64. 10.1016/S0928-0987(03)00038-1 12694888

[30] Zamber CP Lamba JK Yasuda K Farnum J Thummel K Schuetz JD Natural allelic variants of breast cancer resistance protein (BCRP) and their relationship to BCRP expression in human intestine. Pharmacogenetics. (2003) 13(1):19–28. 10.1097/00008571-200301000-00004 12544509

[31] Kim HS Sunwoo YE Ryu JY Kang HJ Jung HE Song IS The effect of ABCG2 V12M, Q141K and Q126X, known functional variants in vitro, on the disposition of lamivudine. Br J Clin Pharmacol. (2007) 64(5):645–54. 10.1111/j.1365-2125.2007.02944.x 17509035 PMC2203270

[32] Imai Y Nakane M Kage K Tsukahara S Ishikawa E Tsuruo T C421a polymorphism in the human breast cancer resistance protein gene is associated with low expression of Q141K protein and low-level drug resistance. Mol Cancer Ther. (2002) 1(8):611–6. 12479221

[33] Kasza I Várady G Andrikovics H Koszarska M Tordai A Scheffer GL Expression levels of the ABCG2 multidrug transporter in human erythrocytes correspond to pharmacologically relevant genetic variations. PLoS One. (2012) 7(11):e48423. 10.1371/journal.pone.0048423 23166586 PMC3499528

[34] Kobayashi D Ieiri I Hirota T Takane H Maegawa S Kigawa J Functional assessment of ABCG2 (BCRP) gene polymorphisms to protein expression in human placenta. Drug Metab Dispos. (2005) 33(1):94–101. 10.1124/dmd.104.001628 15475413

[35] Wu H Liu Y Kang H Xiao Q Yao W Zhao H Genetic variations in ABCG2 gene predict breast carcinoma susceptibility and clinical outcomes after treatment with anthracycline-based chemotherapy. Biomed Res Int. (2015) 2015:279109. 10.1155/2015/279109 26634205 PMC4655035

[36] Leung GK Véniant MM Kim SK Zlot CH Raabe M Björkegren J A deficiency of microsomal triglyceride transfer protein reduces apolipoprotein B secretion. J Biol Chem. (2000) 275(11):7515–20. 10.1074/jbc.275.11.7515 10713055

[37] Oliveira CP Stefano JT Cavaleiro AM Zanella Fortes MA Vieira SM Rodrigues Lima VM Association of polymorphisms of glutamate-cystein ligase and microsomal triglyceride transfer protein genes in non-alcoholic fatty liver disease. J Gastroenterol Hepatol. (2010) 25(2):357–61. 10.1111/j.1440-1746.2009.06001.x 19817962

[38] Ledmyr H, McMahon AD, Ehrenborg E, Nielsen LB, Neville M, Lithell H, et al. The microsomal triglyceride transfer protein gene-493T variant lowers cholesterol but increases the risk of coronary heart disease. Circulation. (2004) 109(19):2279–84. 10.1161/01.CIR.0000130070.96758.7b 15136504

[39] Chen L Yoshino G Maeda E Zeng S. Effect of microsomal triglyceride transfer protein gene polymorphism in the promoter region on dyslipidemia in type 2 diabetic subjects. Chin Med J. (2003) 116(2):215–7. 12775233

[40] El-Koofy NM El-Karaksy HM Mandour IM Anwar GM El-Raziky MS El-Hennawy AM. Genetic polymorphisms in non-alcoholic fatty liver disease in obese Egyptian children. Saudi J Gastroenterol. (2011) 17(4):265–70. 10.4103/1319-3767.82582 21727734 PMC3133985

[41] Wang C, Xie L, Li H, Li Y, Mu D, Zhou R, et al. Associations between ABCG2 gene polymorphisms and isolated septal defects in a Han Chinese population. DNA Cell Biol. (2014) 33(10):689–98. 10.1089/dna.2014.2398 PMC4180187 24979295

[42] Sari FM, Yanar HT, Ozhan G. Investigation of the functional single-nucleotide polymorphisms in the BCRP transporter and susceptibility to colorectal cancer. Biomed Rep. (2015) 3(1):105–9. 10.3892/br.2014.383 PMC4251126 25469257

[43] Kim KA Joo HJ Park JY. ABCG2 polymorphisms, 34G> A and 421C> A in a Korean population: analysis and a comprehensive comparison with other populations. J Clin Pharm Ther. (2010) 35(6):705–12. 10.1111/j.1365-2710.2009.01127.x 21054463

[44] Lundahl B Leren TP Ose L Hamsten A Karpe F. A functional polymorphism in the promoter region of the microsomal triglyceride transfer protein (MTP -493G/T) influences lipoprotein phenotype in familial hypercholesterolemia. Arterioscler Thromb Vasc Biol. (2000) 20(7):1784–8. 10.1161/01.ATV.20.7.1784 10894817

[45] Phillips C Mullan K Owens D Tomkin GH. Microsomal triglyceride transfer protein polymorphisms and lipoprotein levels in type 2 diabetes. QJM. (2004) 97(4):211–8. 10.1093/qjmed/hch040 15028851

